# BAP31 Promotes Epithelial–Mesenchymal Transition Progression Through the Exosomal miR-423-3p/Bim Axis in Colorectal Cancer

**DOI:** 10.3390/ijms26125483

**Published:** 2025-06-07

**Authors:** Changli Wang, Wanting Liu, Sheng Yang, Tianyi Wang, Bing Wang

**Affiliations:** Institute of Biochemistry and Molecular Biology, College of Life and Health Sciences, Northeastern University, Shenyang 110819, China; 1710070@stu.neu.edu.cn (C.W.); 2401474@stu.neu.edu.cn (W.L.); yangsheng@mail.neu.edu.cn (S.Y.)

**Keywords:** BAP31, exosomes, EMT, miR-423-3p, Bim, Alyref

## Abstract

This study explores the regulatory function of BAP31 on exosomal miRNA and its impact on the EMT in CRC. Exosomes from BAP31-OE cells promoted recipient cell migration and triggered the EMT, as indicated by decreased E-cadherin and increased N-cadherin and Vimentin levels. By contrast, exosomes derived from shBAP31 cells were observed to inhibit cell migration and revert EMT markers. The administration of shBAP31 exosomes significantly inhibited tumor growth in vivo. miRNA profiling revealed 76 differentially expressed miRNAs in BAP31-OE exosomes. Six miRNA candidates associated with the EMT were identified in the GEO database, miR-423-3p was identified as a key mediator, the candidates from shBAP31 exosomes exhibited the opposite effect. EMT promotion by miR-423-3p was further evidenced by EMT marker expression, enhanced migratory capacity, and accelerated tumor growth. Sixteen potential target genes were identified through bioinformatics analysis. Bim exhibited significant downregulation by the miR-423-3p mimic. Luciferase reporter assays verified the direct interaction between miR-423-3p and the 3′UTR of Bim. Silencing Bim negated the effects of miR-423-3p. It was also revealed that BAP31 does not influence the total exosomal miRNA content but selectively regulates miR-423-3p, which contains an EXOmotif enriched in BAP31-OE exosomes. Mechanistic studies revealed that BAP31 enhances the expression of the RNA export adaptor Alyref, as validated by qRT-PCR and Western blot analyses. RNA immunoprecipitation assays verified that Alyref binds to miR-423-3p in BAP31-OE cells. Our results reveal that BAP31 facilitates the sorting of exosomal miR-423-3p via Alyref, thereby promoting EMT in CRC through the miR-423-3p/Bim signaling axis. This indicates that BAP31 could be a viable therapeutic target for managing the EMT in CRC.

## 1. Introduction

The epithelial–mesenchymal transition (EMT) is a vital biological process in which epithelial cells lose polarity and cell–cell adhesion while gaining migratory and invasive mesenchymal traits, crucial for cancer metastasis [1]. The EMT can be initiated by various factors, with hypoxia being particularly significant, as it predominantly occurs in the tumor core due to inadequate vascularization [2]. Interestingly, EMT-active cells are often located at the tumor periphery rather than the hypoxic center, implying that hypoxia-induced pro-EMT signals might be conveyed to the outer regions through an unidentified mechanism [3].

B-cell receptor-associated protein 31 (BAP31) is a transmembrane protein predominantly found in the endoplasmic reticulum (ER) [4]. It is extensively expressed in various cell types and acts as a broad-specificity chaperone for membrane protein quality control [5]. Our prior study has shown that BAP31 influences the stability of hypoxia-inducible factor 1α (HIF1α) [6], a crucial transcriptional regulator of hypoxic responses [7]. It has been suggested that HIF1α promotes the EMT under hypoxic conditions [8]. We hypothesize that BAP31 may facilitate the transmission of hypoxia-induced EMT signals from the tumor core to the periphery. Notably, BAP31 overexpression has been observed to induce the EMT, further supporting its potential involvement in this process [9].

Intercellular communication plays a vital role in EMT process. Functioning as an indispensable component for intercellular communication, exosomes have attracted our attention. Exosomes (30–150 nm membrane vesicles) act as carriers of molecular signals between cells through both autocrine and paracrine mechanisms [10]. These vesicles carry bioactive molecules such as proteins, lipids, and nucleic acids (e.g., miRNAs) that can affect recipient cell behavior [11]. One of the key processes influenced by exosomes is the EMT that allows epithelial cells to acquire mesenchymal, migratory, and invasive properties. Considering that EMT in peripheral tumor cells necessitates the activation of multiple signaling pathways, exosomes may serve as effective vehicles for delivering hypoxia-induced pro-EMT factors, such as HIF1α-stabilizing proteins or EMT-promoting RNAs (e.g., LncROR) enhancing the aggressive phenotype of cancer cells under hypoxic conditions [12,13]. In some studies exosomes have been shown to promote EMT in recipient cells by upregulating the miR-193a-3p, miR-210-3p and miR-510 (exosomal RNAs) or TGFβ2 (exosomal protein) affecting signaling factors [14,15].

Based on these observations, we propose that BAP31, potentially through its regulation or interaction with exosomal cargo, may play a pivotal role in conveying EMT signals from the tumor core to the periphery. Investigating this mechanism could provide novel insights into the spatial regulation of the EMT and tumor metastasis.

## 2. Results

### 2.1. Exosomes from Cells with Elevated BAP31 Expression Enhance EMT in Recipient Cells

**Figure 1:**
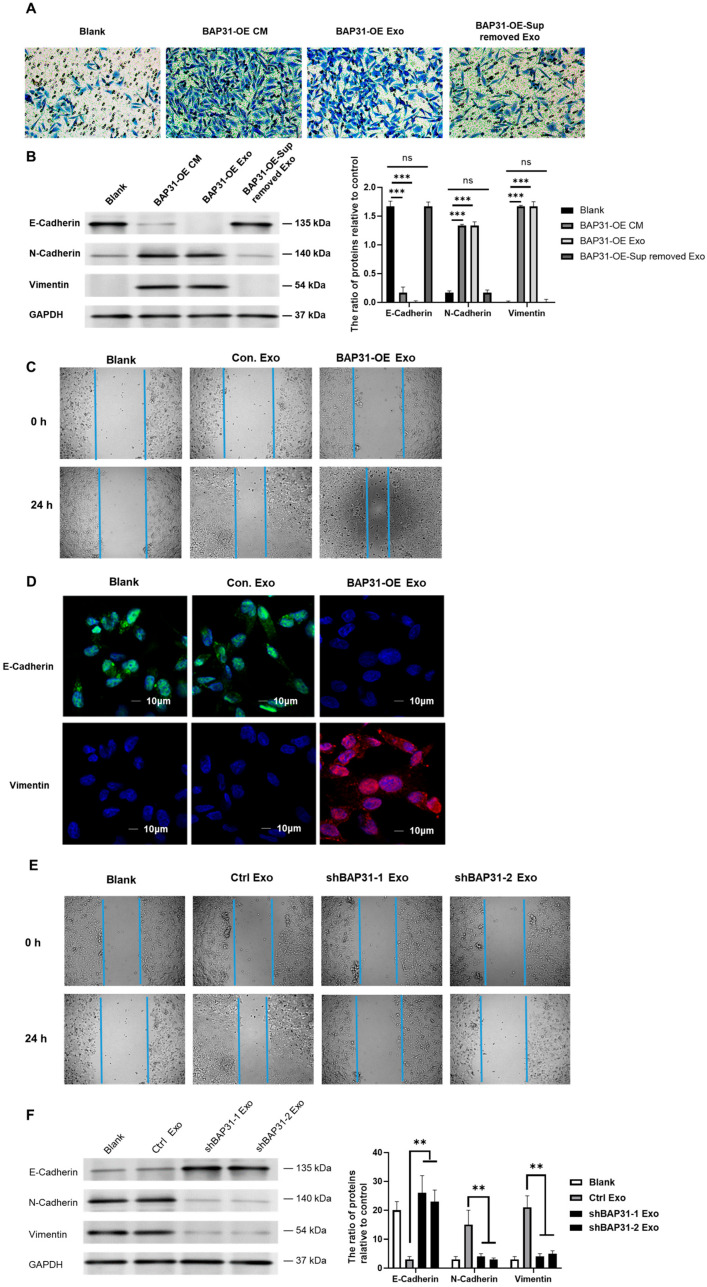
Exosomes originating from BAP31-OE cells facilitate the EMT in recipient cells. (**A**) The Transwell migration assay indicates an increased migratory capacity in recipient cells treated with DMEM (Blank), conditioned medium (CM) and exosomes from BAP31-OE cells in comparison to the Blank group. (**B**) Western blot analysis of EMT markers in recipient cells post-treatment, with GAPDH utilized as a loading control. Densitometric analysis indicated a notable decrease in E-cadherin levels, alongside an elevated expression of N-cadherin and Vimentin (mean ± SEM; *** *p* < 0.001, two-way ANOVA). (**C**) The wound healing assay demonstrated a more rapid closure of gaps in cells treated with BAP31-OE exosomes at 0 and 24 h. (**D**) Immunofluorescence staining of EMT markers (E-cadherin in green, Vimentin in red, and nuclei with DAPI) in recipient cells demonstrated that BAP31-OE exosomes promoted the transition from epithelial to mesenchymal traits. (**E**) Exosomes derived from shBAP31 cells inhibited wound closure in the scratch assay, with quantification confirming reduced migration. (**F**) Western blot analysis of EMT markers in cells treated with shBAP31 exosomes showed restored E-cadherin levels and reduced expression of N-cadherin and Vimentin (mean ± SEM; ** *p* < 0.01, two-way ANOVA). The concentration of exosomes used was 30 μg/mL for each groups.

To elucidate the role of BAP31 in facilitating the EMT within the context of colorectal cancer (CRC), we overexpressed BAP31 in HCT116 cells (Supplementary Figure S1A,B) and harvested the conditioned medium (CM) for subsequent functional assays. Transwell migration assays and Western blot analyses revealed that conditioned media (CM) from BAP31-overexpressing cells (BAP31-OE) significantly promoted recipient cell migration and triggered the EMT, as evidenced by decreased E-cadherin and increased N-cadherin and Vimentin expression. Notably, the removal of exosomes from the CM abrogated this effect, suggesting that the induction of the EMT by BAP31 is contingent upon exosomal secretion (Figure 1A,B). Consistent with these findings, wound healing assays revealed that exosomes from BAP31-overexpressing cells expedited wound closure. The quantitative data confirm the visual observation that BAP31-OE exosomes accelerate wound closure by nearly 4-fold at 24 h (Figure 1C and Figure S9A). Furthermore, confocal microscopy showed that recipient cells incubated with BAP31-OE exosomes exhibited a marked decrease in E-cadherin fluorescence intensity relative to control groups, indicating a loss of epithelial characteristics. In contrast, Vimentin expression was significantly elevated in these cells, corroborating the acquisition of a mesenchymal phenotype (Figure 1D). These results illustrate that exosomes originating from BAP31-overexpressing cells effectively facilitate the EMT in recipient cells by downregulating E-cadherin and upregulating Vimentin.

To ensure the successful isolation of exosomes, we conducted a comprehensive characterization based on morphology, size, and specific markers. Transmission electron microscopy (TEM) analysis revealed that the isolated vesicles exhibited a characteristic cup-shaped morphology, aligning with the anticipated structure of exosomes (Supplementary Figure S2A). Nanoparticle tracking analysis (NTA) revealed that most particles measured between 50 and 150 nm in diameter, supporting their identification as exosomes (Supplementary Figure S2B). Western blot analysis verified the presence of the exosomal markers CD63, TSG101, and Alix, while the negative control (cell lysate) lacked these proteins but expressed GM130, a Golgi apparatus marker (Supplementary Figure S2C). To verify the efficient internalization of the isolated exosomes by recipient cells, we labeled the exosomes with the fluorescent dye PKH67 and incubated them with target cells. Confocal microscopy revealed a notable uptake of PKH67-labeled exosomes, indicated by a punctate green fluorescence in the cytoplasm of recipient cells and reduced by heparin sodium, which is known to inhibit exosome uptake (Supplementary Figure S2D). These results collectively confirm the successful isolation and characterization of exosomes, as well as their efficient internalization by recipient cells.

To further substantiate these findings, we generated BAP31-knockdown HCT116 cells (Supplementary Figure S1C,D) and isolated their exosomes. Treatment with exosomes derived from BAP31-knockdown cells (shBAP31) led to reduced migration rates in recipient cells (Figure 1E) and exerted opposing effects on EMT markers (Figure 1F).

To extend our investigation, we utilized SW480 cells as recipient cells and co-cultured them with exosomes derived from either BAP31-OE or shBAP31 cells. Western blot analysis demonstrated that exposure to these exosomes induced the EMT in SW480 cells, as evidenced by decreased epithelial and increased mesenchymal markers (Supplementary Figure S3A,B).

The results validate the conclusion that exosomes from BAP31-enriched cells are essential in triggering the EMT. Collectively, the results suggest that BAP31 facilitates the EMT in CRC through exosome-mediated intercellular communication, while its knockdown diminishes the pro-metastatic effects on recipient cells.

### 2.2. Exosomes from shBAP31 Cells Suppress Colorectal Cancer Progression

**Figure 2:**
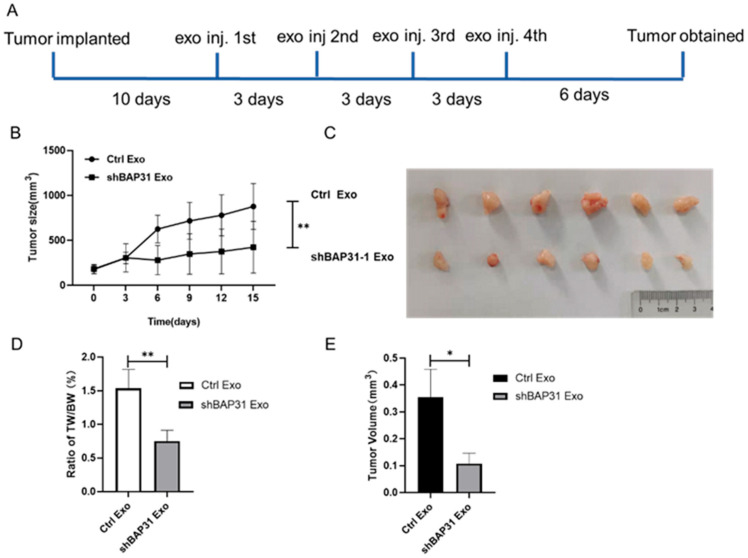
The effects of exosomes derived from shBAP31 on a xenograft tumor model. (**A**) Schematic representation of the experimental design. HCT116 tumor-bearing mice received intratumoral injections of either control exosomes or shBAP31 exosomes (10 μg/100 mm^3^ tumor, administered every 3 days) starting on the 10th day post-transplantation, continuing for a duration of 21 days (n = 6 per group). (**B**) The tumor growth curves demonstrate a notable reduction in tumor volume in the shBAP31 exosome-treated group compared to the control group (mean ± SEM; ** *p* < 0.01, two-way ANOVA). (**C**) Representative images of excised tumors at the study endpoint (day 27) reveal a noticeable reduction in tumor size following treatment with shBAP31 exosomes. (**D**) The shBAP31 exosome-treated group exhibited a significantly lower tumor-to-body weight ratio compared to the control exosome group (mean ± SEM; ** *p* < 0.01, one-way ANOVA). (**E**) The reduction in final tumor volumes corroborated the growth-inhibitory effect of shBAP31 exosomes (mean ± SD; * *p* < 0.05, one-way ANOVA).

To explore the functional role of these exosomes in CRC progression, we established a subcutaneous xenograft model using HCT116 cells in nude mice (Figure 2A). Exosome treatment from shBAP31 cells notably inhibited tumor growth and decreased final tumor volume relative to the control group (Figure 2B–D). Consistently with this, the tumor weight-to-body weight ratio decreased markedly in mice treated with shBAP31 exosomes (Figure 2E), indicating potent antitumor activity in vivo. These findings demonstrate that exosomes derived from shBAP31 cells exhibit antitumor effects by inhibiting primary tumor growth in CRC.

### 2.3. BAP31 Modulates miR-423-3p Within Exosomes Correlating with EMT

**Figure 3:**
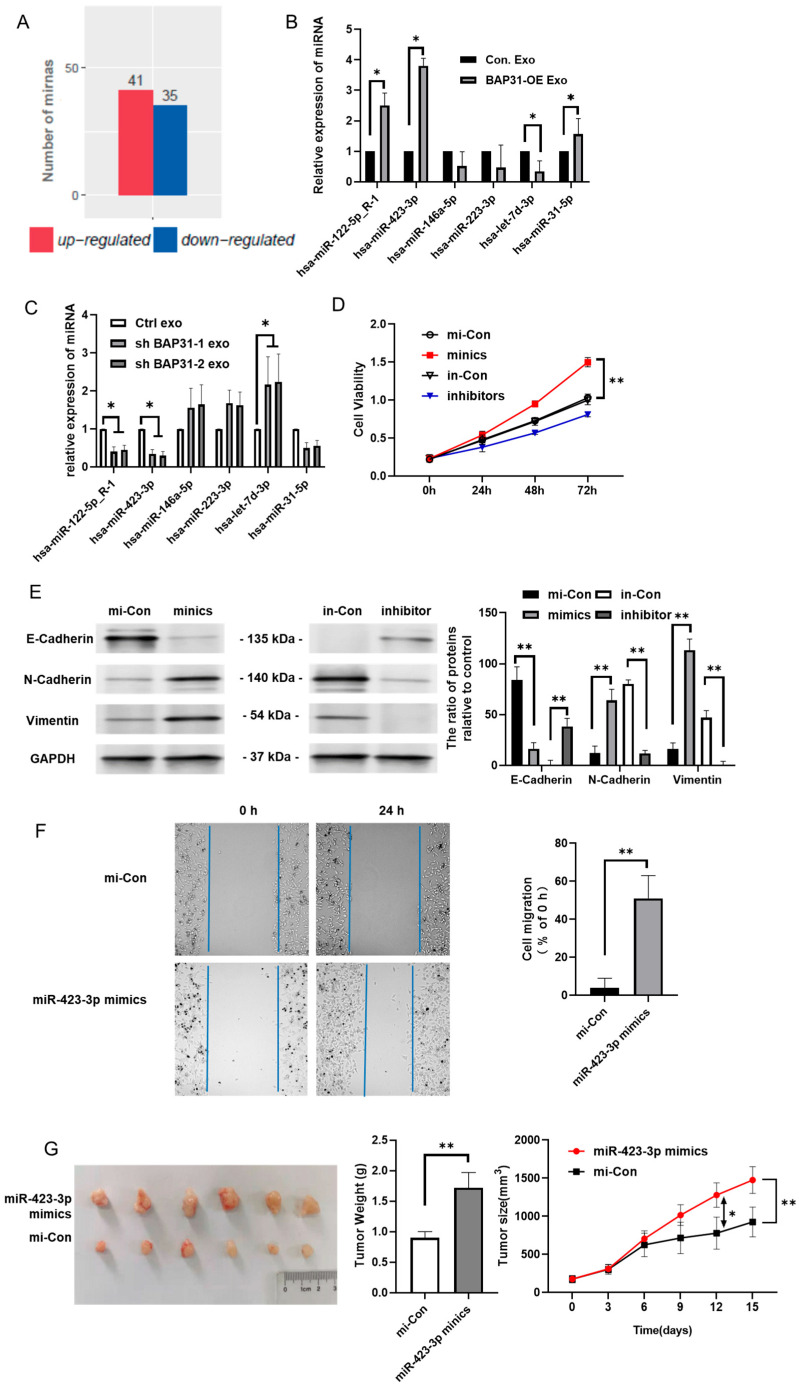
The influence of BAP31 on exosomal miR-423-3p, promoting EMT and tumor progression in vitro and in vivo. (**A**) miRNA profiling analysis identified 76 miRNAs with differential expression in exosomes derived from BAP31-BAP31-OE cells compared to controls, with upregulated miRNAs shown in red and downregulated miRNAs in blue. (**B**) Quantitative reverse-transcription PCR (qRT-PCR) was used to confirm the expression levels of different exosomal miRNAs in BAP31-OE exosomes. The results are shown as mean ± SD, with a sample size of n = 3. Statistical significance was assessed using Student’s *t*-test, with * *p* < 0.05 compared to the control. (**C**) qRT-PCR analysis confirmed exosomal miRNA levels in shBAP31 exosomes, presented as mean ± SD (n = 3), with statistical significance determined using Student’s *t*-test (* *p* < 0.05 compared to control). (**D**) CCK-8 assay demonstrated that a 50 nM miR-423-3p mimic notably enhanced HCT116 cell viability at 48 and 72 h (** *p* < 0.01 compared to mi-Con, analyzed via two-way ANOVA). Statistical significance markers refer to comparisons at the final time point (72 h) (**E**) Western blot analysis of EMT markers in miR-423-3p-treated cells revealed decreased E-cadherin and increased N-cadherin and Vimentin levels, with quantification presented in the right panel (mean ± SD, n = 3; ** *p* < 0.01, one-way ANOVA). (**F**) A wound healing assay confirmed that miR-423-3p enhances cell migration at 0 and 24 h. Statistical analysis in the right panel.; ** *p* < 0.01. (**G**) In a xenograft model, the tumor was significantly increased in the miR-423-3p mimic group compared to the control (* *p* < 0.05, ** *p* < 0.01, n = 6).

Our findings indicate that altering BAP31 expression levels, through either overexpression or knockdown, does not significantly affect the particle size or production quantity of exosomes (Supplementary Figure S4A,B). Considering the rapid onset of the EMT in recipient cells following exosome uptake, we hypothesized that exosomal miRNAs may function as primary signaling mediators. To explore this hypothesis, we conducted miRNA profiling on exosomes derived from BAP31-overexpressing cells, using exosomes from control cells as a negative control. The miRNA sequencing data were provided by Lianchuan Biotechnology Co., Ltd. (Hangzhou, China). The raw sequencing data have been deposited in BioProject database under accession number PRJNA884845. Comparative analysis identified 41 upregulated and 35 downregulated miRNAs in exosomes from BAP31-overexpressing cells compared to controls (Figure 3A).

We analyzed the GSE136194 miRNA dataset from the GEO database (https://www.ncbi.nlm.nih.gov/geo/ accessed on 21 April 2023) to identify EMT-associated miRNAs affected by BAP31, revealing 177 non-coding genes linked to the EMT among 30 samples. By intersecting these with the BAP31-regulated exosomal miRNAs, we selected six highly expressed candidates (hsa-miR-122-5p_R-1, hsa-miR-423-3p, hsa-miR-31-5p, hsa-miR-146a-5p, hsa-miR-223-3p, and hsa-let-7d-3p) for further validation. Quantitative reverse-transcription PCR (qRT-PCR) analysis corroborated the upregulation of hsa-miR-122-5p_R-1, hsa-miR-423-3p, and hsa-miR-31-5p in BAP31-OE exosomes, alongside the downregulation of hsa-let-7d-3p, as illustrated in Figure 3B, aligning with the results obtained from miRNA profiling.

Conversely, qRT-PCR analysis of shBAP31 exosomes revealed inverse expression patterns; hsa-miR-122-5p_R-1 and hsa-miR-423-3p were downregulated, while hsa-let-7d-3p was upregulated, as depicted in Figure 3C. Functional assays using miRNA mimics and inhibitors in recipient cells revealed that miR-423-3p notably enhanced cell proliferation, reduced E-cadherin expression (an epithelial marker), and increased N-cadherin and Vimentin levels (mesenchymal markers), indicating EMT induction, as illustrated in Figure 3D,E. In contrast, hsa-miR-122-5p_R-1 and hsa-let-7d-3p did not exhibit significant effects, as detailed in Supplementary Figure S5A–D. Furthermore, a wound healing assay conducted on recipient cells treated with miR-423-3p mimics demonstrated a significant enhancement in cell migration, reinforcing its role in facilitating the EMT, as presented in Figure 3F. In a xenograft tumor model, the peritumoral injection of miR-423-3p mimics markedly enhanced tumor growth, further confirming its effect (Figure 3G). These findings confirm that exosomal miR-423-3p, regulated by BAP31, is crucial in promoting EMT and tumor progression.

### 2.4. miR-423-3p Enhances EMT Through Its Interaction with Bim

**Figure 4:**
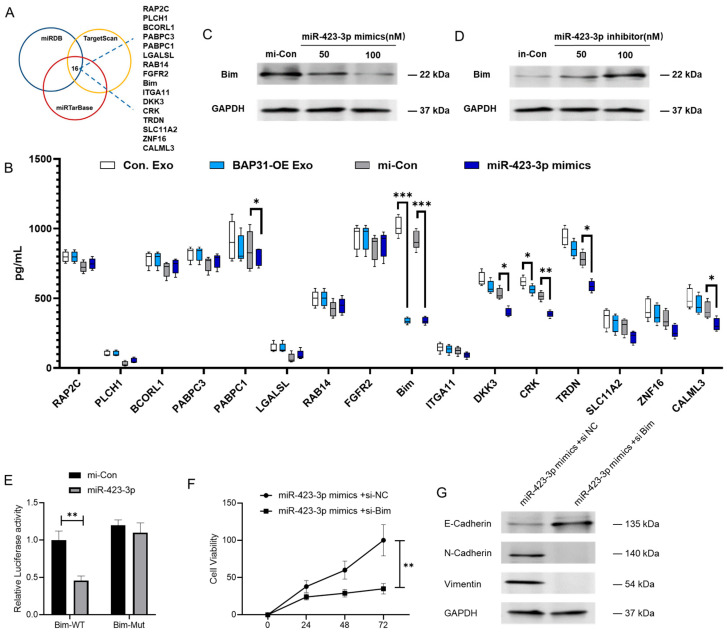
miR-423-3p facilitates the progression of the EMT by directly targeting Bim. (**A**) Schematic diagram generated through bioinformatics prediction using TargetScan, miRDB, and miRTarBase. (https://www.targetscan.org/vert_80/ accessed on 21 June 2023; https://mirdb.org/ accessed on 25 June 2023; https://dianalab.e-ce.uth.gr/tarbasev9 accessed on 21 May 2023) (**B**) ELISA analysis reveals a marked decrease in Bim expression in cells (**B**) exposed to BAP31-OE exosomes or miR-423-3p mimics (50 nM, 48 h), with * *p* < 0.05 ** *p* < 0.01 *** *p* < 0.001 compared to controls, as determined by one-way ANOVA. (**C**) Western blot analysis corroborates the downregulation of Bim by miR-423-3p mimics (48 h). (**D**) An inverse increase in Bim expression is observed following treatment with a miR-423-3p inhibitor (48 h). (**E**) Dual-luciferase assays reveal that miR-423-3p directly binds to the wild-type (WT) Bim 3′UTR, whereas no binding occurs with the mutant (MUT) 3′UTR (** *p* < 0.01 compared to mi-Con, two-way ANOVA). (**F**) CCK-8 assay indicates a reduced pro-survival effect of miR-423-3p when co-transfected with Bim siRNA for 48 h (** *p* < 0.01 compared to si NC). Statistical significance markers refer to comparisons at the final time point (72 h). (**G**) Western blot analysis demonstrates that Bim siRNA treatment reverses miR-423-3p-induced EMT, as indicated by increased E-cadherin levels and decreased N-cadherin and Vimentin expression.

Bioinformatic analyses utilizing miRDB, TargetScan, and miRTarBase revealed 16 potential target genes of miR-423-3p, encoding proteins such as RAP2C, PLCH1, BCORL1, PABPC3, PABPC1, LGALSL, RAB14, FGFR2, Bim, ITGA11, DKK3, CRK, TRDN, SLC11A2, ZNF16, and CALML3 (Figure 4A). We used enzyme-linked immunosorbent assays (ELISA) to assess protein expression changes in cells treated with BAP31-overexpressing exosomes or transfected with a miR-423-3p mimic, evaluating the regulatory effects on these targets. Notably, Bim exhibited the most pronounced and consistent response to both interventions (Figure 4B). Subsequent Western blot analysis further confirmed that Bim protein levels were significantly modulated by miR-423-3p (Figure 4C,D). Additionally, quantitative reverse-transcription PCR (qRT-PCR) revealed that Bim mRNA was downregulated by miR-423-3p across all CRC cell lines (Supplementary Figure S6). Luciferase reporter assays confirmed that miR-423-3p directly interacts with the 3′ untranslated region (UTR) of Bim (Figure 4E). Silencing Bim with siRNA enhanced the proliferation rate of cells affected by miR-423-3p (Figure 4F). The epithelial marker E-cadherin was upregulated, while the mesenchymal markers N-cadherin and Vimentin were downregulated (Figure 4G), suggesting the miR-423-3p/Bim axis may play a role in EMT modulation. Collectively, these findings suggest that Bim serves as a direct and functionally important target of miR-423-3p, thereby influencing its regulatory effects on CRC cell proliferation and EMT dynamics.

### 2.5. BAP31 Promotes the Selective Enrichment of miR-423-3p in Exosomes by Regulating Alyref

The total levels of miRNAs in exosomes were not significantly influenced by the overexpression or knockdown of BAP31 (Supplementary Figure S7), indicating that BAP31 may not play a role in modulating the overall miRNA content. We hypothesized that BAP31 could be involved in the selective loading of miRNAs into exosomes. Notably, we identified that miR-423-3p possesses an EXOmotif (GGCCCC), a specific sequence motif that facilitates the sorting of miRNAs into exosomes [16]. Furthermore, miRNA profiling of exosomes derived from BAP31-overexpressing cells demonstrated that three additional miRNAs containing this EXOmotif (hsa-mir-10396b-p3, hsa-mir-10396a-p3, and PC-3p-105562_4) were also upregulated. To elucidate the underlying mechanism, we investigated the RNA export adaptors Alyref and Fus, which are known to recognize EXOmotifs and mediate the loading of miRNAs into exosomes.

**Figure 5:**
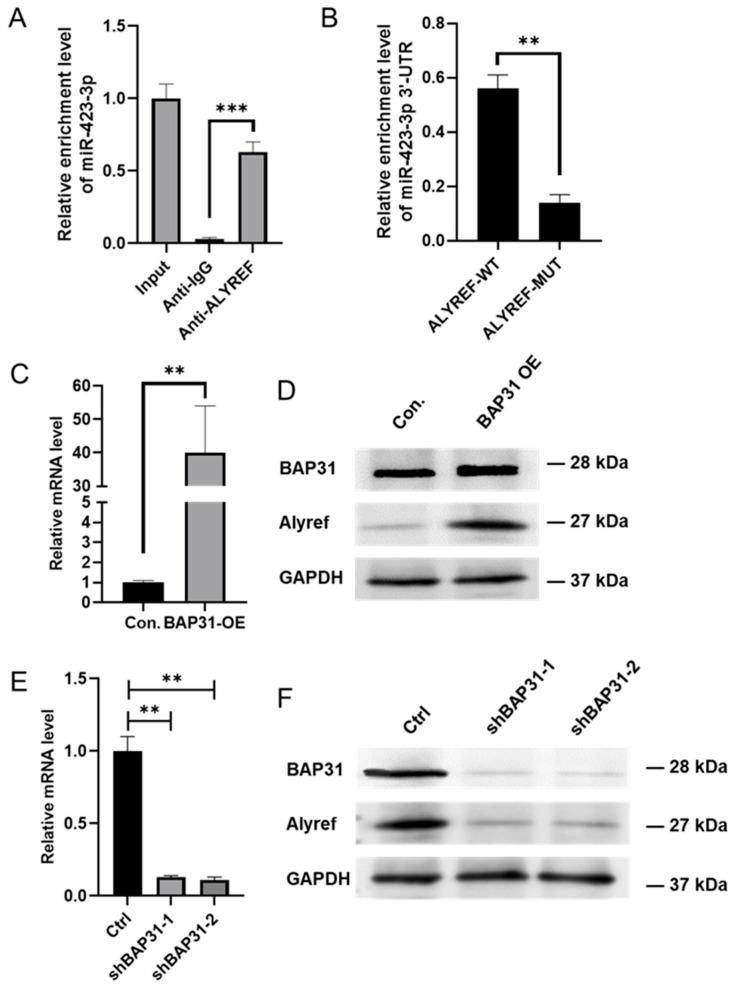
BAP31 facilitates the selective enrichment of miR-423-3p in exosomes through the regulation of Alyref. (**A**) RNA immunoprecipitation (RIP) analysis utilizing an anti-Alyref antibody in BAP31-overexpressing (BAP31-OE) cells demonstrated a significant enrichment of miR-423-3p in ALYREF-RIP samples compared to the IgG control, as determined by qRT-PCR (normalized to input; mean ± SD, *** *p* < 0.001, n = 3; analyzed by Student’s *t*-test). (**B**) RIP assays conducted in HCT116 cells transfected with either wild-type or mutant ALYREF revealed, via qRT-PCR, that the mutation significantly diminished ALYREF’s binding affinity to miR-423-3p (mean ± SD, Student’s *t*-test, ** *p* < 0.01). (**C**) Quantitative reverse-transcription PCR (qRT-PCR) analysis demonstrated significantly increased ALYREF mRNA levels in BAP31-OE cells compared to controls (mean ± SD, n = 3, Student’s *t*-test, ** *p* < 0.01). (**D**) Western blot analysis corroborated the upregulation of Alyref following BAP31 overexpression for 48 h. (**E**) Quantitative reverse-transcription PCR (qRT-PCR) confirmed the elevated ALYREF mRNA levels in BAP31-OE cells (mean ± SD, n = 3, Student’s *t*-test, ** *p* < 0.01 compared to control). (**F**) Western blot analysis demonstrated Alyref downregulation following a 48 h BAP31 knockdown.

RNA immunoprecipitation (RIP) assays revealed that miR-423-3p selectively binds to Alyref in cells overexpressing BAP31 (Figure 5A,B), while no interaction with Fus was observed (Supplementary Figure S8). qRT-PCR and Western blot analyses revealed that Alyref expression increased with BAP31 overexpression and decreased with BAP31 knockdown (Figure 5C–F). The results indicate that BAP31 modulates Alyref expression, affecting the integration of miR-423-3p into exosomes. This mechanism potentially accounts for the observed enrichment of specific miRNAs in exosomes regulated by BAP31.

## 3. Discussion

Exosomes play a pivotal role in intercellular communication within cancerous environments, promoting tumor progression by transferring bioactive molecules, including miRNAs [17]. This study investigates the function of BAP31 in modulating the sorting of exosomal miRNAs and its influence on the EMT in CRC. It identifies a novel BAP31-Alyref-exosomal miR-423-3p-Bim axis involved in CRC metastasis, proposing that BAP31 represents a potential target for therapeutic intervention.

Previous research has established the role of exosomal miRNAs in contributing to the EMT and metastasis across various cancer types [14]. miR-21 and the miR-200 family are recognized as EMT modulators in CRC [18]. However, their levels showed no significant correlation with BAP31 expression (*p* > 0.05 by ANOVA), in contrast to miR-423-3p (*p* < 0.001). Our study extends this understanding by identifying miR-423-3p as a significant player, demonstrating its enrichment in BAP31-OE exosomes and its functional role in promoting the EMT. While miR-423-3p has been previously implicated in hepatocellular carcinoma [19,20], its involvement in CRC-related EMT has not been elucidated. Our findings corroborate existing studies on its oncogenic properties and further identify Bim as a crucial downstream target. Bim, a proapoptotic BH3-only protein, is known to be downregulated in a subset of colorectal cancers. Its interaction with EMT transcription factors like SNAI2 and ZEB1, suggested that Bim plays a significant role in EMT progression by influencing apoptosis and metastasis, and its repression may be a significant step in CRC tumorigenesis [21,22,23]. Although BAP31 has been associated with apoptosis and ER stress [24], its role in the sorting of exosomal miRNAs was previously unrecognized. Our research uncovers a novel function of BAP31 in Alyref-mediated miRNA export, thereby broadening the understanding of its oncogenic mechanisms. Alyref is known to facilitate mRNA nuclear export, yet its role in exosomal miRNA sorting has not been previously explored [16]. Our RIP assays confirm that Alyref binds to miR-423-3p in BAP31-OE cells, suggesting the existence of a BAP31-Alyref axis in the selection of exosomal miRNAs.

The RIP provides valuable insights into the interactions, but RIP identifies associations cannot distinguish the direct binding and indirect complex formation, the functional binding from non-specific aggregation as well as the spatial coordination within ribonucleoprotein complexes. These limitations highlight the need for validation, In vitro reconstitution assays, Single-molecule imaging (e.g., smFISH with protein tagging), CRISPR-based endogenous tagging (e.g., APEX-RIP), Kinetic studies that measure the dynamics of RNA–protein interactions, such as those discussed in the literature, can offer more comprehensive insights into these processes [25,26,27].

While this study offers valuable insights into the role of BAP31 in regulating exosomal miRNA and its impact on the EMT in CRC, several limitations must be acknowledged. In this study, we mainly employed in vitro and xenograft models, which might not completely represent the complexity of the tumor microenvironment. To confirm these findings in a physiologically relevant setting, it is crucial to examine the roles of BAP31 and miR-423-3p within metastasis models. Although Alyref has been identified as a mediator, the precise post-translational regulation of BAP31 on Alyref remains unclear. Our observations indicate that the upregulation of BAP31 leads to an increase in both Alyref mRNA and protein levels. We propose that BAP31 likely regulates Alyref indirectly through intermediate factors rather than via direct interaction. This hypothesis is supported by our previous findings, which demonstrate that BAP31 can promote the proteasomal degradation of p27kip1 [28], suggesting it may similarly influence other protein stabilizers or degraders that affect Alyref. The spatial relationships between Alyref, BAP31, miR-423-3p, and Bim mRNA could be conducted by RNA-FISH co-localization studies. Additionally, proteomic screening may identify potential intermediate regulators involved in this process. Phosphoproteomics or co-immunoprecipitation could elucidate this interaction [29]. Although BAP31 is recognized as a potential therapeutic target for EMT in CRC, further investigation in clinical samples is necessary to validate its therapeutic efficacy. Designing drugs targeting BAP31 represents an alternative avenue for future research.

In conclusion, we offers significant insights into the role of BAP31 in modulating exosomal miRNA content and its influence on EMT and CRC progression. The findings underscore the critical role of miR-423-3p and its target, Bim, in mediating the effects of BAP31 on CRC progression. These findings deepen our understanding of the molecular mechanisms of CRC progression and offer potential avenues for developing new therapeutic strategies targeting the EMT in CRC. Additional mechanistic and clinical studies are required to develop these insights into effective treatments.

## 4. Materials and Methods

### 4.1. Cell Culture and Transfections

The HCT116 and DLD-1 human colorectal cancer cell lines were cultured in Dulbecco’s Modified Eagle’s Medium (DMEM; Gibco, Waltham, MA, USA), whereas SW480 cells were maintained in RPMI 1640 medium (Gibco, Waltham, MA, USA). Both media were enriched with 10% fetal bovine serum, along with 100 U/mL each of streptomycin and penicillin. Cell lines were cultured at 37 °C in a humidified environment with 5% CO_2_.

BAP31-overexpressing (BAP31-OE) and BAP31-knockdown (shBAP31) cell lines were generated as previously described [28]. Conditioned medium (CM) was harvested from HCT116 cells cultured for 48 h in DMEM with reduced fetal bovine serum at 37 °C in a 5% CO_2_ humidified incubator. Cells were cultured in suitable dishes and grown to 80% confluence for transfection experiments. The human ALYREF-pET24b vector and ALYREF mutant (The coding sequence of ALYREF 10 arginine to glutamic acid mutations as R27E, R30E, R34E, R36E, R38E, R224E, R227E, R231E, R233E, and R235E) were also synthesized and introduced into the pET24a vector by Sangon Biotech [30,31]. Plasmid DNA, siRNA, miRNA mimics or inhibitor (GenePharma, Shanghai, China) was transfected using Lipofectamine 3000 (Thermo Fisher Scientific, Waltham, MA, USA) following the manufacturer’s guidelines. Supplementary Table S2. provides a list of all synthetic miRNA mimics and inhibitor used in this study.

### 4.2. Cell Counting Assay

Cells were plated in 96-well plates at 5000 cells per well in 100 µL of culture medium and incubated for 24 h to allow for attachment. After exposure to experimental conditions, 10 µL of CCK-8 solution was added to each well. The plates were then incubated at 37 °C for 1 h. Absorbance at 450 nm was recorded using a Biotek Synergy H1 microplate reader (BioTek Instruments, Winooski, VT, USA).

### 4.3. Confocal Laser Scanning

The membrane dye PKH67 (Sigma, St. Louis, Mo, USA) was employed to fluorescently label the exosomes. The recipient cells were co-cultured with the exosomes for 24 h. Before sealing the coverslips, the nuclei were stained with Antifade Mounting Medium containing DAPI. Fluorescence signals were examined with a Leica SP5 confocal laser scanning microscope (Leica Microsystems, Wetzlar, Germany).

### 4.4. Exosome Isolation

Exosomes were isolated from cell culture supernatants through a series of differential ultracentrifugation steps. Cell culture supernatants were first centrifuged at 200× *g* for 10 min to remove cells and debris. The supernatant was centrifuged again at 10,000× *g* for 30 min to eliminate larger vesicles and apoptotic bodies. The resulting supernatant was then ultracentrifuged at 100,000× *g* for 90 min to pellet the exosomes. The exosome pellet underwent a PBS wash followed by re-centrifugation at 100,000× *g* for 90 min to remove remaining contaminants. The exosome pellet was resuspended in PBS and stored at −80 °C for future use [32].

### 4.5. Nanoparticle Tracking Analysis

An aliquot of the isolated exosome solution was transferred to a disposable cuvette for analysis. The sample underwent size distribution analysis using a Nano-ZS90 laser nanoparticle size distribution instrument (Malvern Instruments, Worcestershire, UK), with settings configured in accordance with the manufacturer’s instructions.

### 4.6. Transmission Electron Microscopy

Exosome samples were preserved using 2% glutaraldehyde and underwent dehydration via a graded ethanol series. The samples were embedded in resin, sliced into ultrathin sections, and placed on carbon-coated grids. High-resolution TEM imaging was performed with a JEOL JEM-1400 electron microscope at 120 kV (JEOL, Tokyo, Japan). The image of the exosome morphology was captured on a charge-coupled device (CCD) camera using transmitted electrons.

### 4.7. Transwell Assay

Transwell inserts equipped with polycarbonate membranes (Corning) were utilized in this study. Cells were suspended in a serum-free medium with 0.1% bovine serum albumin (BSA) and seeded at 5 × 10^4^ cells per insert in the upper chamber. The lower chamber contained either conditioned medium (CM) or another liquid acting as a chemoattractant. The cells were incubated for 48 h at 37 °C in a 5% CO_2_ atmosphere. Cells that migrated to the lower surface were fixed with 4% paraformaldehyde and stained with 0.1% crystal violet. The quantification of migrated cells was performed by counting cells in five randomly selected fields.

### 4.8. Wound Healing Assay

Cells were cultured to confluence in 6-well plates. A linear wound was introduced into the monolayer by scratching with a 200 µL pipette tip. Detached cells and debris were removed by washing with PBS, after which the cells were cultured in medium with or without the specified treatment. Wound images were taken at 0 and 24 h with a light microscope and digital camera. Wound closure was assessed by measuring the width at various points and determining the percentage of closure over time. The wound closure was calculated using ImageJ software version 1.54f. Results were presented as the percentage of the 0 h.

### 4.9. RT-PCR Analysis

Total RNA was isolated employing the TRIzol reagent (Invitrogen, Waltham, MA, USA). miRNA quantification was performed using reverse transcription with stem–loop primers and 100 ng of total RNA. Quantitative real-time PCR (qRT-PCR) was conducted with the Mir-X miRNA qRT-PCR TB Green^®^ Kit (Takara, Dalian, China) on a Bio-Rad CFX96 Real-Time PCR Detection System. Primer sequences were specifically designed using Primer-BLAST (https://www.ncbi.nlm.nih.gov/tools/primer-blast/index.cgi?LINK_LOC=BlastHome accessed on 3 January 2023). Gene expression levels were quantified using the 2^−ΔΔCt^ method, normalized against U6 or GAPDH reference genes. Supplementary Table S1 provides a comprehensive list of all primers used in this study.

### 4.10. Western Blot Analysis

Protein samples were extracted using RIPA lysis buffer (Sigma-Aldrich, St. Louis, MO, USA) with added protease inhibitor cocktails (MCE) to prevent degradation. Protein concentration was measured using the BCA Protein Assay Kit (Thermo Fisher Scientific, Waltham, MA, USA) following the manufacturer’s instructions. Samples were separated using 8–12% SDS-PAGE and transferred to 0.2 μm PVDF membranes. Membranes were blocked using 5% skimmed milk powder in TBST for one hour, followed by overnight incubation at 4 °C with primary antibodies diluted in 3% BSA (see Supplementary Table S3 for details). This was followed by incubation with the appropriate secondary antibodies. Membranes were ultimately washed with TBST and visualized using High-sig ECL Western blotting Substrate (Tanon, Shanghai, China). Band intensities were quantified using ImageJ software by measuring the integrated density of the bands. Target protein expression levels were normalized to a housekeeping protein to correct for loading variations.

### 4.11. ELISA

Receptor cell samples were appropriately diluted with a calibrator diluent and measured using the ELISA universal kit (Sangon, Shanghai, China) according to the protocol instructions. Antibodies were conjugated with HRP using the Lightning-Link HRP Labeling Kit (Abcam, Cambridge, UK). Optical density measurements were taken with a Synergy H1 microplate reader (BioTek Instruments, Winooski, VT, USA) and normalized against the standard curve.

### 4.12. Dual-Luciferase Activity Assay

Cells were co-transfected with miRNA mimics, a firefly luciferase reporter plasmid incorporating either wild-type (WT) or mutated 3′UTR sequences, and a Renilla luciferase control plasmid. After a 48 h incubation period, the cells were lysed using Passive Lysis Buffer (Promega, Madison, WI, USA). Luciferase activity was quantified using the Dual-Luciferase Reporter Gene Assay Kit (Beyotime, Shanghai, China) according to the manufacturer’s instructions, with measurements taken on a Synergy H1 microplate reader (Biotek, Winooski, VT, USA). Relative luciferase activity was determined by normalizing and comparing the values across different experimental groups.

### 4.13. RNA Immunoprecipitation Assay

1 × 10^6^ recipient cells (HCT116 cells) were lysed using lysis buffer (Thermo Fisher Scientific, Waltham, MA, USA) supplemented with RNase-free protease and phosphatase inhibitors (Roche, Basel, Switzerland). The lysates were incubated overnight at 4 °C with 20 µL magnetic beads conjugated to antibodies specific for the RNA-binding proteins Alyref or Fus or with control IgG antibodies (Santa Cruz Biotechnology, Dallas, TX, USA). Following incubation, the beads were washed with lysis buffer three times and then digested with Proteinase K (Sigma-Aldrich, St. Louis, MO, USA) for 1 h. Then, they underwent RNA purification using TRIzol Reagent. The RNAs was resuspended in RNase-free water and then reverse-transcribed and analyzed using qRT-PCR to quantify specific RNA targets. The primers used for analysis are listed in Supplementary Table S1.

### 4.14. Animal Experiments

The animal experiments adhered to institutional guidelines and were approved by the Institutional Animal Care and Use Committee (IACUC). BALB/C mice, aged 6–8 weeks, were obtained from Huafukang Biotechnology and housed in an SPF animal facility. Cells were prepared at 2 × 10^6^ cells/mL in antibiotic-containing phosphate-buffered saline (PBS) and injected subcutaneously (0.1 mL per site) into the flanks of mice in an SPF environment. Tumor growth was tracked with caliper measurements, and tumor volume was determined using the formula: (length × width^2^)/2. The Animal Care and Use Committee of Northeastern University, China, reviewed and approved the animal study protocol.

Isolated exosomes were resuspended in PBS and administered through intratumoral injection for treatment. The injection volume was adjusted based on tumor size, typically ranging from 10 to 20 µL per injection. Mice underwent exosome treatment every three days, totaling four sessions.

### 4.15. Statistical Analysis

GraphPad Prism 8 software was used for statistical analyses. Each experiment was conducted at least three times, including independent biological replicates. Data are presented as mean ± standard deviation (SD). Student’s *t*-test was used for comparing two groups. *p*-values below 0.05 were considered statistically significant.

## Data Availability

The data that support the findings of this study are available from the corresponding author upon reasonable request.

## Supplementary Materials

The following supporting information can be downloaded at: https://www.mdpi.com/article/10.3390/ijms26125483/s1.

## References

[1] Nieto M.A., Huang R.Y.-J., Jackson R.A., Thiery J.P. (2016). EMT: 2016. Cell.

[2] Harris A.L. (2002). Hypoxia—A key regulatory factor in tumour growth. Nat. Rev. Cancer.

[3] Krishnamachary B., Zagzag D., Nagasawa H., Rainey K., Okuyama H., Baek J.H., Semenza G.L. (2006). Hypoxia-inducible factor-1-dependent repression of E-cadherin in von Hippel-Lindau tumor suppressor-null renal cell carcinoma mediated by TCF3, ZFHX1A, and ZFHX1B. Cancer Res..

[4] Schamel W.W., Kuppig S., Becker B., Gimborn K., Hauri H.P., Reth M. (2003). A high-molecular-weight complex of membrane proteins BAP29/BAP31 is involved in the retention of membrane-bound IgD in the endoplasmic reticulum. Proc. Natl. Acad. Sci. USA.

[5] Wang B., Heath-Engel H., Zhang D., Nguyen N., Thomas D.Y., Hanrahan J.W., Shore G.C. (2008). BAP31 interacts with Sec61 translocons and promotes retrotranslocation of CFTRDeltaF508 via the derlin-1 complex. Cell.

[6] Namusamba M., Wu Y., Yang J., Zhang Q., Wang C., Wang T., Wang B. (2024). BAP31 Promotes Angiogenesis via Galectin-3 Upregulation in Neuroblastoma. Int. J. Mol. Sci..

[7] Majmundar A.J., Wong W.J., Simon M.C. (2010). Hypoxia-inducible factors and the response to hypoxic stress. Mol. Cell.

[8] Yang M.-H., Wu M.-Z., Chiou S.-H., Chen P.-M., Chang S.-Y., Liu C.-J., Teng S.-C., Wu K.-J. (2008). Direct regulation of TWIST by HIF-1alpha promotes metastasis. Nat. Cell Biol..

[9] Li T., Hao Z., Tang Z., Li C., Cheng L., Wang T., Zhu X., He Y., Huang Y., Wang B. (2022). BAP31 Regulates Wnt Signaling to Modulate Cell Migration in Lung Cancer. Front. Oncol..

[10] Théry C., Zitvogel L., Amigorena S. (2002). Exosomes: Composition, biogenesis and function. Nat. Rev. Immunol..

[11] Valadi H., Ekström K., Bossios A., Sjöstrand M., Lee J.J., Lötvall J.O. (2007). Exosome-mediated transfer of mRNAs and microRNAs is a novel mechanism of genetic exchange between cells. Nat. Cell Biol..

[12] Hoshino A., Costa-Silva B., Shen T.-L., Rodrigues G., Hashimoto A., Tesic Mark M., Molina H., Kohsaka S., Di Giannatale A., Ceder S. (2015). Tumour exosome integrins determine organotropic metastasis. Nature.

[13] Wang H., Min J., Xu C., Liu Y., Yu Z., Gong A., Xu M. (2023). Hypoxia-elicited Exosomes Promote the Chemoresistance of Pancreatic Cancer Cells by Transferring LncROR via Hippo Signaling. J. Cancer.

[14] Zhang X., Sai B., Wang F., Wang L., Wang Y., Zheng L., Li G., Tang J., Xiang J. (2019). Hypoxic BMSC-derived exosomal miRNAs promote metastasis of lung cancer cells via STAT3-induced EMT. Mol. Cancer.

[15] Qin W., Tsukasaki Y., Dasgupta S., Mukhopadhyay N., Ikebe M., Sauter E.R. (2016). Exosomes in Human Breast Milk Promote EMT. Clin. Cancer Res..

[16] Garcia-Martin R., Wang G., Brandão B.B., Zanotto T.M., Shah S., Kumar Patel S., Schilling B., Kahn C.R. (2021). MicroRNA sequence codes for small extracellular vesicle release and cellular retention. Nature.

[17] Kalluri R., LeBleu V.S. (2020). The biology, function, and biomedical applications of exosomes. Science.

[18] Kang E., Jung S.C., Nam S.K., Park Y., Seo S.H., Park K.U., Oh H.-K., Kim D.W., Kang S.-B., Lee H.S. (2022). Tissue miR-200c-3p and circulating miR-1290 as potential prognostic biomarkers for colorectal cancer. Sci. Rep..

[19] Wong V.C.-L., Wong M.-I., Lam C.-T., Lung M.L., Lam K.-O., Lee V.H.-F. (2021). Hallmark microRNA signature in liquid biopsy identifies hepatocellular carcinoma and differentiates it from liver metastasis. J. Cancer.

[20] Lin J., Huang S., Wu S., Ding J., Zhao Y., Liang L., Tian Q., Zha R., Zhan R., He X. (2011). MicroRNA-423 promotes cell growth and regulates G_1_/S transition by targeting p21Cip1/Waf1 in hepatocellular carcinoma. Carcinogenesis.

[21] Merino D., Best S.A., Asselin-Labat M.-L., Vaillant F., Pal B., Dickins R.A., Anderson R.L., Strasser A., Bouillet P., Lindeman G.J. (2015). Pro-apoptotic Bim suppresses breast tumor cell metastasis and is a target gene of SNAI2. Oncogene.

[22] Inoue-Yamauchi A., Oda H. (2020). EMT-inducing transcription factor ZEB1-associated resistance to the BCL-2/BCL-XL inhibitor is overcome by BIM upregulation in ovarian clear cell carcinoma cells. Biochem. Biophys. Res. Commun..

[23] Greenhough A., Wallam C.A., Hicks D.J., Moorghen M., Williams A.C., Paraskeva C. (2010). The proapoptotic BH3-only protein Bim is downregulated in a subset of colorectal cancers and is repressed by antiapoptotic COX-2/PGE_2_ signalling in colorectal adenoma cells. Oncogene.

[24] Rosati E., Sabatini R., Rampino G., De Falco F., Di Ianni M., Falzetti F., Fettucciari K., Bartoli A., Screpanti I., Marconi P. (2010). Novel targets for endoplasmic reticulum stress-induced apoptosis in B-CLL. Blood.

[25] Licatalosi D.D., Ye X., Jankowsky E. (2020). Approaches for measuring the dynamics of RNA-protein interactions. Wiley Interdiscip. Rev. RNA.

[26] Lee F.C.Y., Ule J. (2018). Advances in CLIP Technologies for Studies of Protein-RNA Interactions. Mol. Cell.

[27] Xiang J.S., Schafer D.M., Rothamel K.L., Yeo G.W. (2024). Decoding protein-RNA interactions using CLIP-based methodologies. Nat. Rev. Genet..

[28] Chen J., Guo H., Jiang H., Namusamba M., Wang C., Lan T., Wang T., Wang B. (2019). A BAP31 intrabody induces gastric cancer cell death by inhibiting p27 proteasome degradation. Int. J. Cancer.

[29] Yang X., Yang Y., Sun B.-F., Chen Y.-S., Xu J.-W., Lai W.-Y., Li A., Wang X., Bhattarai D.P., Xiao W. (2017). 5-methylcytosine promotes mRNA export—NSUN2 as the methyltransferase and ALYREF as an mC reader. Cell Res..

[30] Chong P.A., Vernon R.M., Forman-Kay J.D. (2018). RGG/RG motif regions in RNA binding and phase separation. Mol. Biol..

[31] Bhandari J., Guillén-Mendoza C., Banks K., Eliaz L., Southwell S., Eyaa D., Luna R., Aguilera A., Xue X. (2024). The molecular chaperone ALYREF promotes R-loop resolution and maintains genome stability. J. Biol. Chem..

[32] Théry C., Witwer K.W., Aikawa E., Alcaraz M.J., Anderson J.D., Andriantsitohaina R., Antoniou A., Arab T., Archer F., Atkin-Smith G.K. (2018). Minimal information for studies of extracellular vesicles 2018 (MISEV2018): A position statement of the International Society for Extracellular Vesicles and update of the MISEV2014 guidelines. J. Extracell. Vesicles.

