# Supplementary material for: BAP31 Promotes Epithelial–Mesenchymal Transition Progression Through the Exosomal miR-423-3p/Bim Axis in Colorectal Cancer
Source: Int J Mol Sci. 2025 Jun 7;26(12):5483. doi: 10.3390/ijms26125483 (PMC12193162; doi:10.3390/ijms26125483)
Supplement: Supplementary file 1 [file ijms-26-05483-s001.zip › Supplementary Figure S1.pdf]

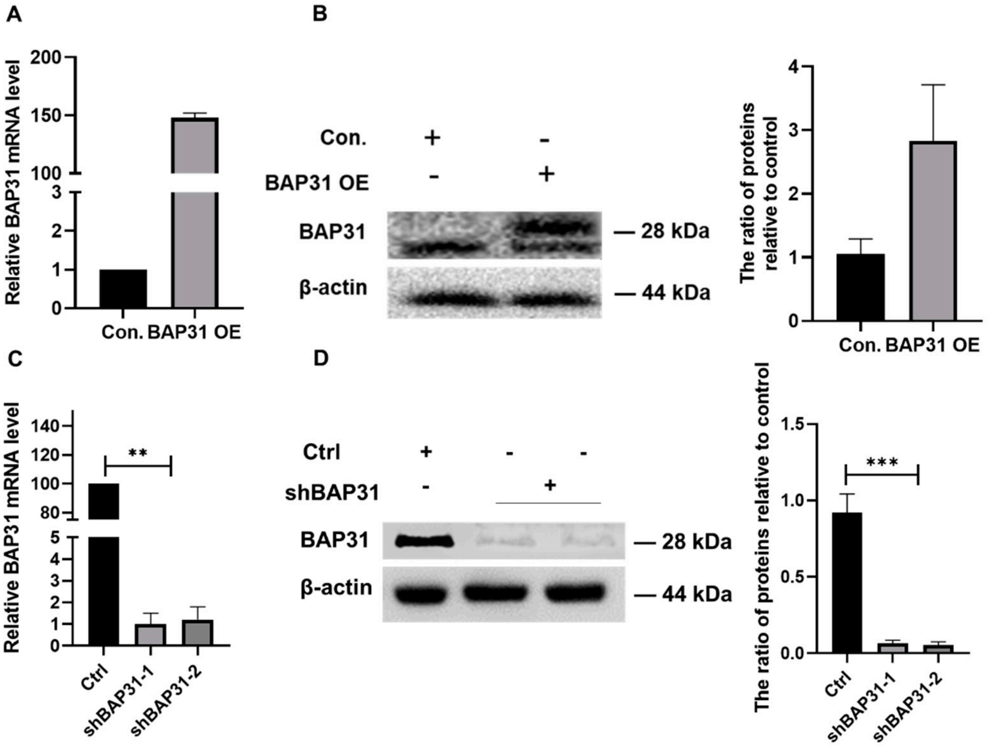

Supplementary Figure 1. Establishment of BAP31-overexpressing (BAP31-OE) and BAP31-knockdown (shBAP31) cell lines.

(A) RT-qPCR analysis was utilized to assess BAP31 mRNA expression levels in both control (Con.) and BAP31-OE cell lines. Expression data were normalized to GAPDH and are presented as mean ± standard deviation (SD) from three independent experiments (n=3). Statistical significance was determined as **p < 0.01 compared to the control.

(B) Western blot analysis of BAP31 protein expression in BAP31-OE cell lines. with β-actin serving as a loading control. Band intensity quantification (right panel) was performed using ImageJ software, and the data are expressed as mean ± SD from three independent experiments, with ***p < 0.001 versus control.

(C) RT-qPCR analysis of BAP31 mRNA expression levels was performed in control (Ctrl) and shBAP31 cell lines. Data normalization was conducted using GAPDH, and results are shown as mean ± SD (n=3), with **p < 0.01 compared to the control.

(D) Western blot analysis assessed BAP31 protein expression in shBAP31 cell lines, using β-actin as a loading control. Band intensity quantification (right panel) was performed using ImageJ software, and results are presented as mean ± SD from three independent experiments, with ***p < 0.001 compared to the control.
