# Supplementary material for: BAP31 Promotes Epithelial–Mesenchymal Transition Progression Through the Exosomal miR-423-3p/Bim Axis in Colorectal Cancer
Source: Int J Mol Sci. 2025 Jun 7;26(12):5483. doi: 10.3390/ijms26125483 (PMC12193162; doi:10.3390/ijms26125483)
Supplement: Supplementary file 1 [file ijms-26-05483-s001.zip › Supplementary Figure S2.pdf]

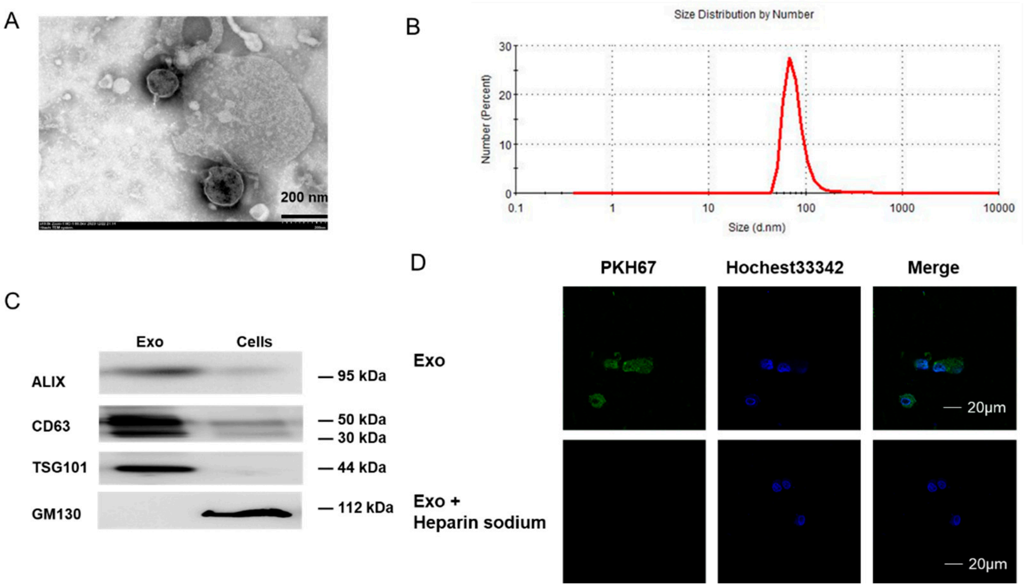


**Supplementary Figure 2 Characterization and functional validation of isolated exosomes.**

(A) Transmission electron microscopy (TEM) revealed typical cup-shaped morphology of purified exosomes (scale bar: 200 nm).

(B) Nanoparticle tracking analysis (NTA) demonstrated exosome size distribution with a peak diameter of 80 nm.

(C) Western blot confirmed enrichment of exosomal markers (CD63, TSG101, Alix) and absence of negative control GM130 (Golgi marker).

(D) Confocal microscopy showed efficient uptake of PKH67-labeled exosomes (green) by recipient cells after 20 h incubation (nuclei: Hochest 33342, blue,scale bar: 20 μm).

All experiments were performed with exosomes isolated from three independent biological replicates.
