# Supplementary material for: BAP31 Promotes Epithelial–Mesenchymal Transition Progression Through the Exosomal miR-423-3p/Bim Axis in Colorectal Cancer
Source: Int J Mol Sci. 2025 Jun 7;26(12):5483. doi: 10.3390/ijms26125483 (PMC12193162; doi:10.3390/ijms26125483)
Supplement: Supplementary file 1 [file ijms-26-05483-s001.zip › Supplementary Figure S3.pdf]

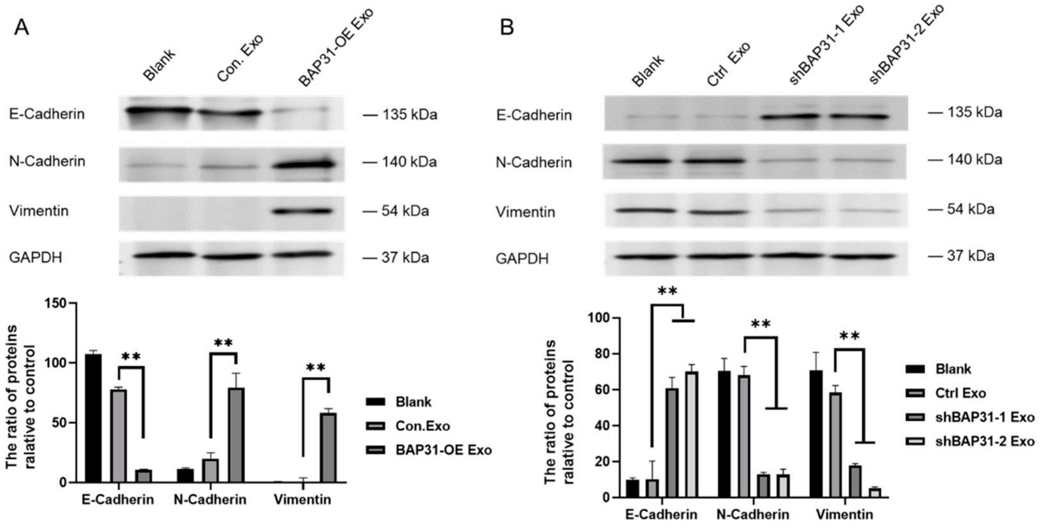

Supplementary Figure 3 Effects of BAP31-modulated exosomes on EMT-related molecules in SW480 cells.

(A) Western blot analysis of EMT markers (E-cadherin, N-cadherin, Vimentin) in SW480 cells treated with exosomes derived from BAP31- BAP31-OE cells. GAPDH served as loading control. Quantification (down panel) showed significant downregulation of E-cadherin and upregulation of N-cadherin/Vimentin (mean ± SD, n=3; ***p < 0.001 vs. control exosomes, one-way ANOVA).

(B) Western blot of EMT markers in SW480 cells treated with exosomes from shBAP31 cells. Densitometric analysis (down panel) demonstrated reversal of EMT phenotype with elevated E-cadherin and reduced N-cadherin/Vimentin expression (mean ± SD, n=3; ***p < 0.001 vs. control, one-way ANOVA).

Statistical analysis was performed using GraphPad Prism 8.0 with Tukey's post-hoc test.
