# Supplementary material for: BAP31 Promotes Epithelial–Mesenchymal Transition Progression Through the Exosomal miR-423-3p/Bim Axis in Colorectal Cancer
Source: Int J Mol Sci. 2025 Jun 7;26(12):5483. doi: 10.3390/ijms26125483 (PMC12193162; doi:10.3390/ijms26125483)
Supplement: Supplementary file 1 [file ijms-26-05483-s001.zip › Supplementary Figure S4.pdf]

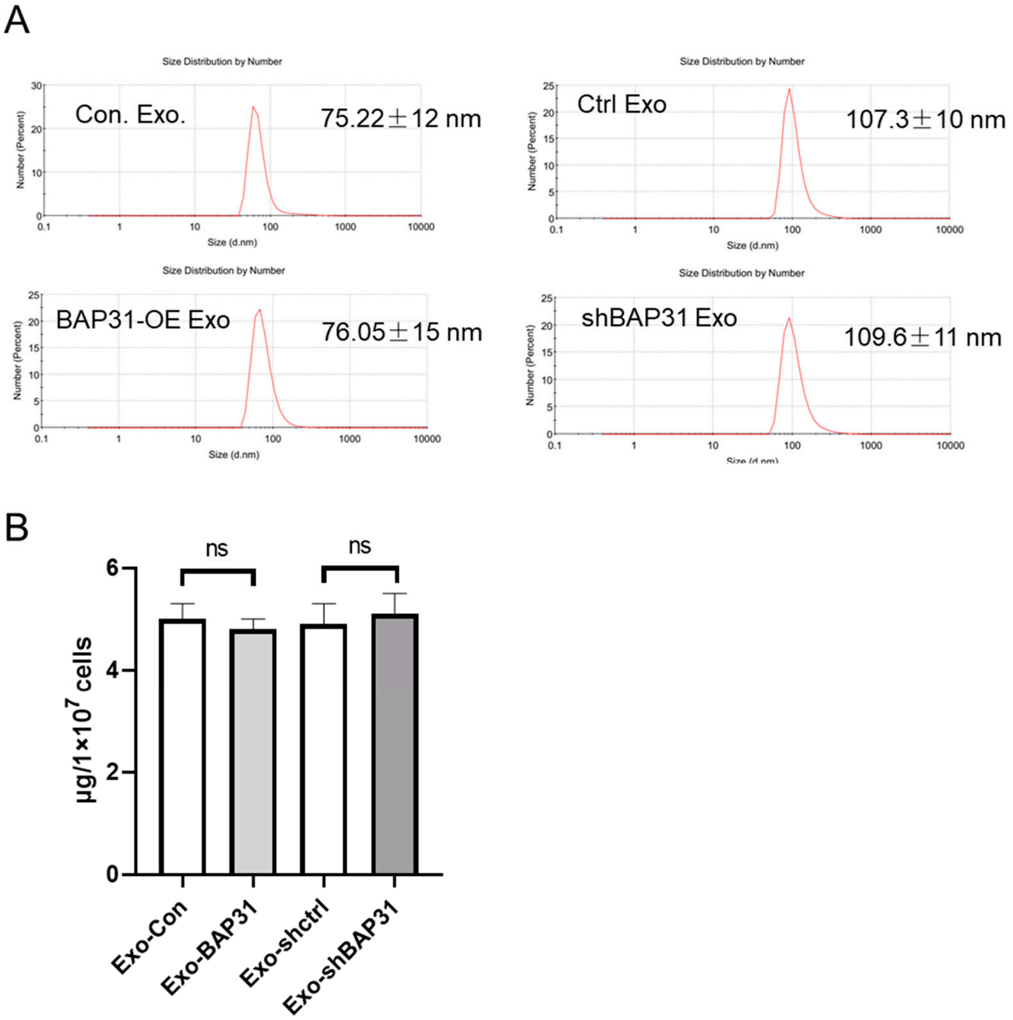

Supplementary Figure 4 BAP31 expression does not alter exosome biophysical properties or production yield.

(A) Nanoparticle tracking analysis (NTA) revealed similar size distribution profiles of exosomes derived from control (Con.), BAP31 overexpressing (BAP31-OE), control (Ctrl), and BAP31 knockdown (shBAP31) cells, with peak diameters of 75.22±12 nm, 76.05±15 nm, 107.3±10 nm, and 109.3±11 nm, respectively.

(B) Quantitative analysis of exosome secretion indicated no significant differences in particle yield per 10^7 cells among the groups (mean±SD, n=3, NS: not significant, $p>0.05$). All measurements were conducted using consistent isolation protocols and instrument calibration.
