# Supplementary material for: BAP31 Promotes Epithelial–Mesenchymal Transition Progression Through the Exosomal miR-423-3p/Bim Axis in Colorectal Cancer
Source: Int J Mol Sci. 2025 Jun 7;26(12):5483. doi: 10.3390/ijms26125483 (PMC12193162; doi:10.3390/ijms26125483)
Supplement: Supplementary file 1 [file ijms-26-05483-s001.zip › Supplementary Figure S5.pdf]

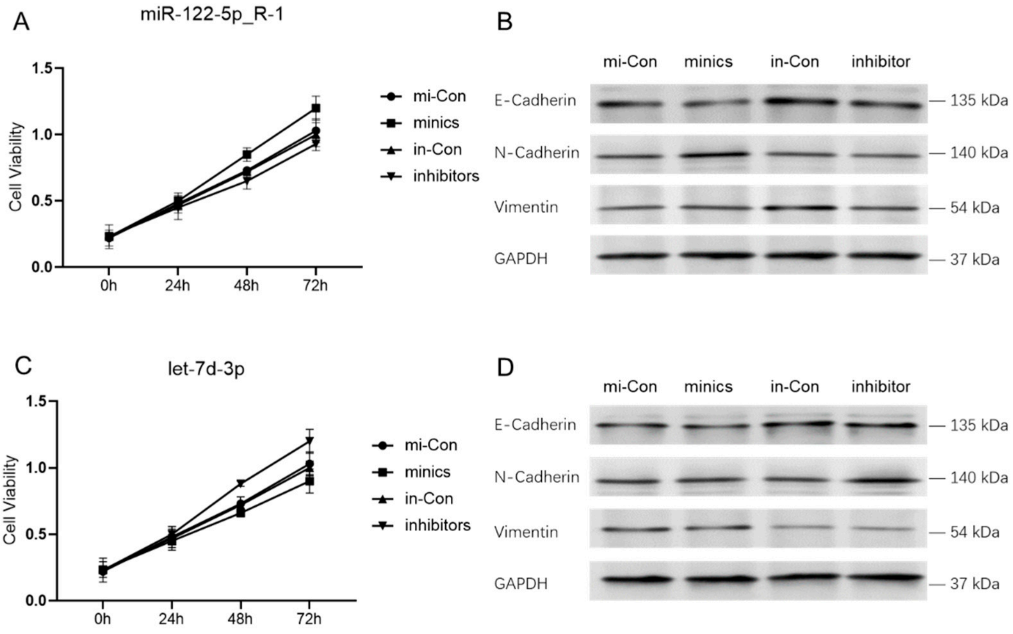


**Supplementary Figure 5 Functional validation of hsa-miR-122-5p_R-1 and hsa-let-7d-3p in cell viability and EMT regulation**

(A) The CCK-8 assay indicated that neither hsa-miR-122-5p_R-1 mimics nor the hsa-miR-122-5p_R-1 inhibitor (50 nM) significantly affected the viability of HCT116 cells after 24, 48, and 72 hours of treatment, when compared to the mimic control(mi-Con) or inhibitor control(in-Con) (mean ± SD, analyzed by two-way ANOVA).

(B) Western blot analysis demonstrated that the expression levels of epithelial-mesenchymal transition (EMT) markers, including E-cadherin, N-cadherin, and Vimentin, remained unchanged in HCT116 cells treated with either hsa-miR-122-5p_R-1 mimics or the inhibitor, using GAPDH as a loading control.

(C) Similarly, the administration of hsa-let-7d-3p mimics or inhibitor (50 nM) did not exhibit cytotoxic effects in CCK-8 viability assays conducted at the same three time points, (mean ± SD; not significant compared to mimic control(mi-Con) or inhibitor control(in-Con), analyzed by two-way ANOVA).

(D) The treatment with hsa-let-7d-3p mimics or inhibitor did not result in any modulation of EMT marker expression, as determined by Western blot analysis.
