# Supplementary material for: BAP31 Promotes Epithelial–Mesenchymal Transition Progression Through the Exosomal miR-423-3p/Bim Axis in Colorectal Cancer
Source: Int J Mol Sci. 2025 Jun 7;26(12):5483. doi: 10.3390/ijms26125483 (PMC12193162; doi:10.3390/ijms26125483)
Supplement: Supplementary file 1 [file ijms-26-05483-s001.zip › Supplementary Figure S6.pdf]

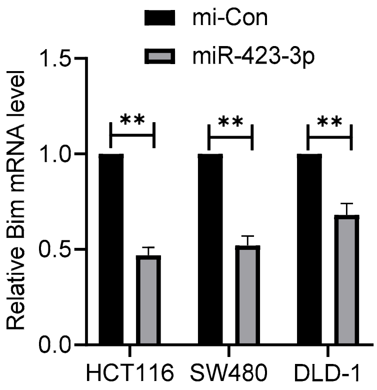

Supplementary Figure 6 miR-423-3p regulatedr Bim mRNA across various CRC cell lines.

qRT-PCR analysis revealed a consistent downregulation of Bim mRNA expression in several CRC cell lines, namely HCT116, SW480, and DLD-1, following transfection with a miR-423-3p mimic (50 nM for 48 hours), as compared to a negative control miRNA (mi-Con). The data were normalized to GAPDH expression and are presented as mean fold-change ± standard deviation (SD), with three biological replicates per cell line. Statistical analysis using two-way ANOVA indicated a highly significant difference (**$p<0.01$) compared to the mi-Con group.
