# Supplementary material for: BAP31 Promotes Epithelial–Mesenchymal Transition Progression Through the Exosomal miR-423-3p/Bim Axis in Colorectal Cancer
Source: Int J Mol Sci. 2025 Jun 7;26(12):5483. doi: 10.3390/ijms26125483 (PMC12193162; doi:10.3390/ijms26125483)
Supplement: Supplementary file 1 [file ijms-26-05483-s001.zip › Supplementary Figure S7.pdf]

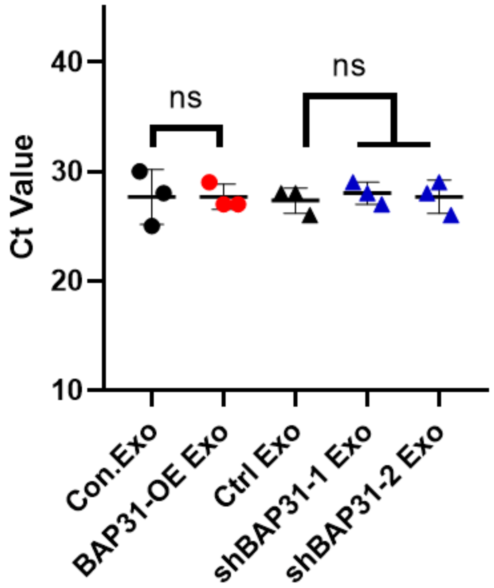


**Supplementary Figure 7 The expression of BAP31 does not affect the overall miRNA content in secreted exosomes.**

The cycle threshold values (Ct values) derived from RT-PCR reactions are shown for U6 RNA to confirm the expression levels of total miRNAs in exosomes derived from Con Exo, BAP31-OE Exo, Ctrl Exo, and shBAP31 Exo.

The results are shown as mean ± SEM, with a sample size of n=3. Statistical significance was assessed using Student's t-test, with $*p<0.01$ com-pared to the control.
