# Supplementary material for: BAP31 Promotes Epithelial–Mesenchymal Transition Progression Through the Exosomal miR-423-3p/Bim Axis in Colorectal Cancer
Source: Int J Mol Sci. 2025 Jun 7;26(12):5483. doi: 10.3390/ijms26125483 (PMC12193162; doi:10.3390/ijms26125483)
Supplement: Supplementary file 1 [file ijms-26-05483-s001.zip › Supplementary Figure S8.pdf]

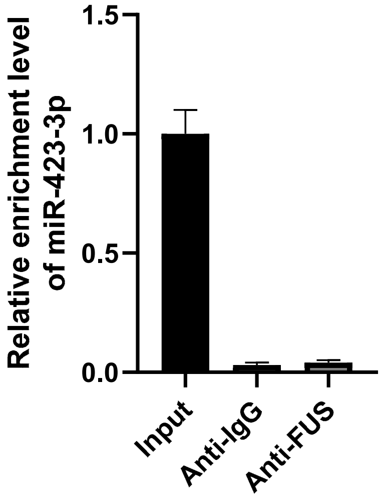

Supplementary Figure 8 BAP31-mediated sorting of miR-423-3p into exosomes is independent of Fus interaction.

RNA immunoprecipitation (RIP) analysis using anti-Fus antibody in BAP31-overexpressing (BAP31-OE) cells. qRT-PCR analysis showing no significant enrichment of miR-423-3p in Fus-RIP samples compared to IgG control (normalized to input; mean ± SD, n=3; by Student's t-test)
