# Supplementary material for: BAP31 Promotes Epithelial–Mesenchymal Transition Progression Through the Exosomal miR-423-3p/Bim Axis in Colorectal Cancer
Source: Int J Mol Sci. 2025 Jun 7;26(12):5483. doi: 10.3390/ijms26125483 (PMC12193162; doi:10.3390/ijms26125483)
Supplement: Supplementary file 1 [file ijms-26-05483-s001.zip › Supplementary Figure S9.pdf]

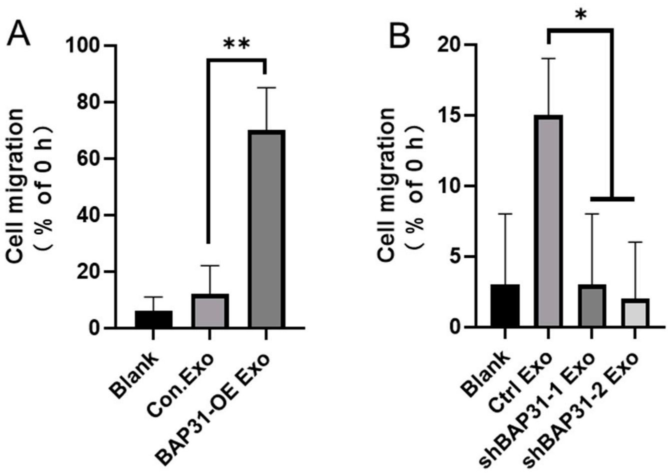

Supplementary Figure 9. Statistical analysis of wound-healing assay.

(A) The wound healing assay demonstrated a more rapid closure of gaps in cells treated with BAP31-OE exosomes at 0 and 24 hours. Statistical analysis of wound-healing assay for Figure 1C. **P < 0.01.

(B) Exosomes derived from shBAP31 cells inhibited wound closure in the scratch assay, with quantification confirming reduced migration. Statistical analysis of wound-healing assay for Figure 1E.; **P < 0.05.
