# Supplementary material for: BAP31 Promotes Epithelial–Mesenchymal Transition Progression Through the Exosomal miR-423-3p/Bim Axis in Colorectal Cancer
Source: Int J Mol Sci. 2025 Jun 7;26(12):5483. doi: 10.3390/ijms26125483 (PMC12193162; doi:10.3390/ijms26125483)
Supplement: Supplementary file 1 [file ijms-26-05483-s001.zip › Supplementary Table S1.pdf]

Supplementary Table 1 Primer sequences used in this study.

Gene	Primer sequences (5'-3')
miR-423-3p	Stem Loop: CTCAACTGGTGTCGTGGAGTCGGCAATTCAGTTGAGCTGAGGGG
	Forward: TCGGCAGGAGCTCGGTCTGAGGCCCCTCAG
miR-122-5p_R-1	Stem Loop: CTCAACTGGTGTCGTGGAGTCGGCAATTCAGTTGAGAAACACCA
	Forward: TCGGCAGGTGGAGTGTGACAATGGTGTTT
miR-146a-5p	Stem Loop: CTCAACTGGTGTCGTGGAGTCGGCAATTCAGTTGAG AACCCATG
	Forward: 5′-GCCGAGTGAGAACTGAATTCCATGGGTT
miR-223-3p	Stem Loop: CTCAACTGGTGTCGTGGAGTCGGCAATTCAGTTGAGTGGGGTAT
	Forward: TCGGCAGGTGTCAGTTTGTCAAATACCCCA
miR-31-5p	Stem Loop: CTCAACTGGTGTCGTGGAGTCGGCAATTCAGTTGAG AGCTATGC
	Forward: GCCGAGAGGCAAGATGCTGGCATAGCT
let-7d-3p	Stem Loop: CTCAACTGGTGTCGTGGAGTCGGCAATTCAGTTGAGAGAAAGGC
	Forward: GCCGAGCTATACGACCTGCTGCCTTTCT
U6	Forward: CTCGCTTCGGCAGCACA
	Reverse: AACGCTTCACGAATTTGCGT
Universal	Reverse: CTCAACTGGTGTCGTGGA
ALYREF	Forward: GCAGGCCAAAACAACTTCCC
	Reverse: AGTTCCTGAATATCGGCGTCT
GAPDH	Forward: GACAGTCAGCCGCATCTTCT
	Reverse: TTAAAAGCAGCCCTGGTGAC
