# Supplementary material for: BAP31 Promotes Epithelial–Mesenchymal Transition Progression Through the Exosomal miR-423-3p/Bim Axis in Colorectal Cancer
Source: Int J Mol Sci. 2025 Jun 7;26(12):5483. doi: 10.3390/ijms26125483 (PMC12193162; doi:10.3390/ijms26125483)
Supplement: Supplementary file 1 [file ijms-26-05483-s001.zip › Supplementary Table S2.pdf]

Supplementary Table 2. Sequences of synthetic miRNA mimics and inhibitor.

Primer name	Primer sequence (5′→3′)
mi-Con.	UUCUCCGAACGUGUCACGUUU
in-Con.	CAGUACUUUUGUGUAGUACAA
miR-423-3p mimics	Sence: AGCUCGGUCUGAGGCCCCUCAGU
	Anti-sence: UCGAGCCAGACUCCGGGGAGUCA
miR-423-3p inhibitor	ACUGAGGGGCCUCAGACCGAGCUU
miR-122-5p_R-1 mimics	Sence: TGGAGTGTGACAATGGTGTTT
	Anti-sence: ACCUCACACUGUUACCACAAA
miR-122-5p_R-1 inhibitor	AAACACCAUUGUCACACUCCA
let-7d-3p mimics	Sence: CUAUACGACCUGCUGCCUUUCU
	Anti-sence: GAUAUGCUGGACGACGGAAAGA
let-7d-3p inhibitor	AGAAAGGCAGCAGGUCCGUAUAG

miRNA mimics and inhibitor were chemically synthesized and HPLC-purified by GenePharma (Shanghai, China)
