# Supplementary material for: BAP31 Promotes Epithelial–Mesenchymal Transition Progression Through the Exosomal miR-423-3p/Bim Axis in Colorectal Cancer
Source: Int J Mol Sci. 2025 Jun 7;26(12):5483. doi: 10.3390/ijms26125483 (PMC12193162; doi:10.3390/ijms26125483)
Supplement: Supplementary file 1 [file ijms-26-05483-s001.zip › Supplementary Table S3.pdf]

Supplementary Table 3 List of antibodies.

Names	Manufacturer
E-Cadherin Rabbit mAb	Abcam
N-Cadherin Rabbit mAb	Abcam
Vimentin Mouse mAb	Abcam
Alix Rabbit pAb	Wanlei
CD63 Rabbit pAb	Wanlei
TSG101 Rabbit pAb	Wanlei
GM130 Rabbit pAb	Wanlei
RAP2C Rabbit pAb	Merck
PLCH1 Rabbit pAb	Abbexa
BCORL1 Rabbit pAb	Abbexa
PABPC3 Rabbit pAb	Abbexa
PABPC1 Rabbit pAb	Abbexa
LGALSL Rabbit pAb	Abbexa
RAB14 Rabbit pAb	Abbexa
FGFR2 Rabbit pAb	Abbexa
Bim Rabbit mAb/ Rabbit pAb	Abcam/ Abbexa
ITGA11	Abbexa
DKK3	Abbexa
CRK	Abbexa
TRDN	Abbexa
SLC11A2	Abbexa
ZNF16	Abbexa
CALML3	Abbexa
Alyref Rabbit mAb	Abcam
Fus Rabbit mAb	Abcam
Goat Anti-Rabbit IgG H&L (HRP)	Abcam
Goat Anti-Rabbit IgG H&L (Alexa Fluor® 488)	Abcam
Goat Anti-Mouse IgG H&L (Alexa Fluor® 647)	Abcam
Goat Anti-Mouse IgG H&L (HRP)	Abcam
